# Phase I study of enzastaurin and bevacizumab in patients with advanced cancer: safety, efficacy and pharmacokinetics

**DOI:** 10.1007/s10637-012-9850-6

**Published:** 2012-07-06

**Authors:** Nwabundo Nwankwo, Zhe Zhang, Ting Wang, Connie Collins, Lee Resta, Sabine Ermisch, Jeannette Day, Rodney Decker, Lori Kornberg, Steven Nicol, Donald Thornton, Deborah K. Armstrong, Michael A. Carducci

**Affiliations:** 1 Kimmel Cancer Center at Johns Hopkins, Baltimore, MD USA; 2 Eli Lilly and Company, Indianapolis, IN USA; 3 PharmaNet/i3, an inVentiv Health company, Princeton, NJ USA; 4 Chemical Therapeutics Program, Kimmel Cancer Center at Johns Hopkins, CRB 1 M59, 1650 Orleans Street, Baltimore, MD 21231-1000 USA

**Keywords:** Enzastaurin, Bevacizumab, Phase I, Advanced cancer, Ovarian cancer

## Abstract

*Purpose* Given distinct mechanism of actions of enzastaurin and bevacizumab, preclinical studies suggest enhanced antitumor activity in combination. This phase I study assessed the combination’s safety and efficacy. *Patients and methods *Six advanced cancer patients could be enrolled in each of 11 cohorts. Patients received an enzastaurin loading dose. Oral enzastaurin (500 mg once daily [QD], 250 mg twice daily [BID], 375 mg BID, 500 mg BID, and 750 mg BID) was escalated in each cohort in combination with bevacizumab dosed at 5 mg/kg every 2 weeks, 10 mg/kg every 2 weeks, or 15 mg/kg every 3 weeks until a dose-limiting toxicity (DLT) occurred in 2 of 6 patients in any cohort. *Results* Sixty-seven patients (31, ovarian cancer [ovcar]) were evaluable for safety and efficacy. Six treatment-related DLTs occurred: grade 3 fatigue (*n* = 4), grade 4 cerebral hemorrhage, and grade 3 elevated aspartate transaminase. Common drug-related toxicities included change in color of urine and stool, fatigue, pain, diarrhea, and nausea. The maximum tolerated dose of enzastaurin was 750 mg BID in combination with any tested bevacizumab dose/schedule. Overall response rate was 19.4 % (32.3 % ovcar). Median time to progression was 3.7 months (95 % confidence interval [CI], 2.7–5.5), with 8.3 months (95 % CI, 3.7–11.1) in ovcar. Overall, 35.9 % (50.4 % ovcar) of patients remained without disease progression after 6 months. *Conclusion* The recommended phase II doses of enzastaurin were 500 mg QD up to 500 mg BID with any tested dose/schedule of bevacizumab. This combination demonstrated encouraging clinical activity, particularly in ovcar.

**Electronic supplementary material:**

The online version of this article (doi:10.1007/s10637-012-9850-6) contains supplementary material, which is available to authorized users.

## Introduction

Enzastaurin HCL (enzastaurin, LY317615) was developed as a selective PKCβ inhibitor [1, 2]. In cultured cancer cells, enzastaurin has antiproliferative and antiapoptotic activities [2]. Enzastaurin has antiangiogenic activity [3]. Enzastaurin also inhibits the AKT pathway with reduced phosphorylation of glycogen synthase kinase 3β (GSK3-β) and AKT [2].

**Table 1:** Baseline patient demographics

Characteristic	All Patients (*N* = 67)	Ovarian Cancer Subset (*n* = 31)
Median age (range), years	61.3 (23.3 to 84.6)	60.5 (23.3 to 73.7)
Gender		
Female	49 (73.1)	31 (100.0)
Male	18 (26.9)	–
Origin		
Caucasian	59 (88.1)	26 (83.9)
African-American	4 (6.0)	1 (3.2)
Asian	1 (1.5)	1 (3.2)
Not reported/unknown	3 (4.5)	3 (9.7)
ECOG Performance Status, n (%)		
0	45 (67.2)	25 (80.7)
1	22 (32.8)	6 (19.3)
Tumor type, n (%)		
Bladder	3 (4.5)	–
Breast	3 (4.5)	–
Esophagus	2 (3.0)	–
Kidney	2 (3.0)	–
Ovarian	31 (46.3)	31 (100.0)
Parotid	2 (3.0)	–
Peritoneal	5 (7.5)	–
Prostate	6 (9.0)	–
Uterine papillary	2 (3.0)	–
Other	11 (16.4)	–
Prior therapies, n (%)		
At least 1 prior	66 (98.5)	30 (96.8)
Surgery	52 (77.6)	25 (80.7)
Radiotherapy	16 (23.9)	1 (3.2)
Chemotherapy	61 (91.0)	30 (96.8)
Immunotherapy	6 (9.0)	1 (3.2)
Hormonal	6 (9.0)	3 (9.7)
Supportive	1 (1.5)	0 (0.0)

*ECOG* Eastern Cooperative Oncology Group

**Table 2:** Pharmacokinetics of enzastaurin and bevacizumab

C_av,ss_ of Enzastaurin and LY326020 (Geometric Mean [CV %])*					
Bevacizumab Dose	5 mg/kg	10 mg/kg		15 mg/kg	
500 mg QD					
N	3	1^a^		2^a^	
Enzastaurin	673 (25)	393 (NC)		1200, 678 (NC)	
LY326020	596 (37)	744 (NC)		609, 718 (NC)	
250 mg BID					
N	–	6		4	
Enzastaurin	–	557 (117)		503 (78)	
LY326020	–	527 (178)		812 (61)	
375 mg BID					
N	–	4		6	
Enzastaurin	–	1030 (66)		1110 (112)	
LY326020	–	1200 (22)		1060 (45)	
500 mg BID					
N	–	4		4	
Enzastaurin	–	1400 (77)		1460 (125)	
LY326020	–	990 (66)^b^		1220 (35)	
750 mg BID					
N	–	–		4	
Enzastaurin	–	–		2660 (118)	
LY326020	–	–		1420 (58)^b^	
Bevacizumab Exposure (Geometric Mean [CV %])**					
Enzastaurin Dosing Regimen	500 mg QD	250 mg BID	375 mg BID	500 mg BID	750 mg BID
Bevacizumab 5 mg/kg					
N	6	–	–	–	–
AUC_(0-∞)_	848 (63)	–	–	–	–
Bevacizumab 10 mg/kg					
N	6	6	3	5	2^a^
AUC_(0-∞)_	2360 (17)	1770 (43)	1740 (5)	1770 (22)	1570, 1600 (NC)
Bevacizumab 15 mg/kg					
N	5	5	6	5	3
AUC_(0-∞)_	2250 (42)	2590 (45)	1640 (26)	2080 (25)	1820 (19)

*AUC*_*(0-∞)*_ area under the concentration-versus-time curve from zero to infinity; *BID* twice daily; *C*_*av,ss*_ average drug concentration at steady state; *CV* coefficient of variation; *N* number of patients with calculable estimates; *NC* not calculable; *QD* once daily; (–) no data in group

* C_av,ss_ (nmol/L) of enzastaurin and LY326020 from cycle 2, day 1 following once- or twice-daily dosing of enzastaurin with bevacizumab

**Bevacizumab AUC_(0-∞)_ (μg·day/mL) estimates from cycle 1, day 1 following an intravenous infusion of bevacizumab with enzastaurin

^a^Insufficient data to calculate mean, individual parameter estimates listed

^b^ *N* = 3

**Table 3:** Summary of efficacy

Variable	All Patients (*N* = 67)			Ovarian Cancer (*N* = 31)		
	All	QD	BID	All	QD	BID
	*N* = 67	*n* = 18	*n* = 49	*N* = 31	*n* = 7	*n* = 24
Response rate, n (%)^a^	13 (19.4)	6 (33.3)	7 (14.3)	10 (32.3)	4 (57.1)	6 (25.0)
95 % CI^b^	10.8 to 30.9	13.3 to 59.0	5.9 to 27.2	16.7 to 51.4	18.4 to 90.1	9.8 to 46.7
Best overall response, n (%)						
Complete response	6 (9.0)	5 (27.8)	1 (2.0)	4 (12.9)	3 (42.9)	1 (4.2)
Partial response	7 (10.4)	1 (5.6)	6 (12.2)	6 (19.4)	1 (14.3)	5 (20.8)
Stable disease	29 (43.3)	5 (27.8)	24 (49.0)	14 (45.2)	1 (14.3)	13 (54.2)
Progressive disease	13 (19.4)	7 (38.9)	6 (12.2)	3 (9.7)	2 (28.6)	1 (4.2)
Not assessed	12 (17.9)	0.0 (0.0)	12 (24.5)	4 (12.9)	0.0 (0.0)	4 (16.7)
Median duration of response (range), mo^c^	6.7 (1.9 to 29.6)	10.2 (1.9 to 29.6)	5.6 (2.8 to 25.2)	6.1 (1.9 to 29.6)	7.4 (1.9 to 29.6)	5.2 (2.8 to 25.2)
Median duration of stable disease (95 % CI), mo^d^	3.7 (2.8 to 4.1)	2.8 –	3.7 (2.8 to 4.1)	3.9 (3.7 to 5.5)	–	4.0 (3.7 to 11.3)
6-month rate of duration (95 % CI)	27.3 (11.2 to 43.4)	–	24.2 (7.3 to 41.1)	23.4 (3.4 to 43.4)	–	25.2 (3.8 to 46.6)
Median time to disease progression or death (95 % CI), mo^e^	3.7 (2.7 to 5.5)	2.8 (1.9 to 7.0)	3.7 (2.7 to 5.5)	8.3 (3.7 to 11.1)	5.5 –	8.3 (3.7 to 11.3)
6-month TTP rate (95 % CI)	35.9 (23.9 to 47.9)	38.9 (17.9 to 59.9)	34.4 (20.3 to 48.5)	50.4 (32.4 to 68.4)	–	52.6 (32.0 to 73.2)

*CI* confidence interval; *CR* complete response; *PD* progressive disease; *PR* partial response; *RECIST* Response Evaluation Criteria in Solid Tumors; *SD* stable disease

^a^Defined as the proportion of patients achieving a CR plus PR using RECIST version 1.0

^b^95 % CI based on exact binomial probabilities

^c^Measured from the date that measurement criteria are met for CR or PR until the first date of documented PD. Duration of response was censored at the date of the last assessment visit for responders with no evidence of PD

^d^Measured from the date of the first dose until the first date of PD. Duration of SD was censored at the date of the last assessment visit for patients with SD with no evidence of PD

^e^Defined as the time from the date of the first enzastaurin or bevacizumab dose to the first date of PD. Time to disease progression was censored at the date of the last assessment visit for patients with no evidence of PD. Estimated using the Kaplan-Meier method

Vascular endothelial growth factor (VEGF) is a regulator of blood vessel growth [4]. Bevacizumab is a humanized anti-VEGF monoclonal antibody [4]. Because bevacizumab and enzastaurin mechanisms of action did not appear to overlap, we hypothesized that the combination might have additive or synergistic effects on tumors.

This study explored whether enzastaurin could be safely combined with bevacizumab in patients with advanced or metastatic cancer and evaluated preliminary antitumor activity of the combination. This study characterized enzastaurin pharmacokinetics (PK) when administered with bevacizumab. Enzastaurin was administered as in previous phase I studies and at higher doses and in different schedules than were previously used [5–9]. Based on known activity of bevacizumab in ovarian cancer (ovcar) [10], this study enrolled a large proportion of patients with the disease.

## Patients and methods

### Eligibility

Key eligibility criteria included histologic or cytologic diagnosis of advanced or metastatic cancer for which no preferable therapy existed; ≥18 years of age; Eastern Cooperative Oncology Group (ECOG) performance status of 0 to 2; measurable or nonmeasurable disease as defined by Response Evaluation Criteria in Solid Tumors (RECIST version 1.0); [11] and an estimated life expectancy of ≥12 weeks.

Key exclusion criteria included inability to swallow tablets; inability to discontinue phenytoin, carbamazepine, and phenobarbital; clinically significant cardiac disease; central nervous system metastases or tumor; evidence of bleeding diathesis or coagulopathy, or requirement for concurrent systemic anticoagulation; and history of major surgery, open biopsy, or significant traumatic injury within 28 days of treatment.

This study was conducted in accordance with the declaration of Helsinki and applicable good clinical practice guidelines. Human investigations were performed after approval by a local Human Investigations Committee and in accordance with an assurance filed with and approved by the Department of Health and Human Services. Written informed consent was obtained according to federal and local guidelines.

### Study design and treatment

This was a single-center, open-label, nonrandomized, dose-escalating phase I trial. The objectives were to: determine the recommended phase II doses (RP2D) of enzastaurin and bevacizumab; characterize toxicities; document antitumor activity; evaluate PK; and assess phosphorylated GSK3-β (pGSK3-β) as a biomarker of enzastaurin. Because pharmacokinetic exposure variation was expected, cohorts of 6 were utilized. Planned enrollment was 66 patients.

**Fig. 1:**
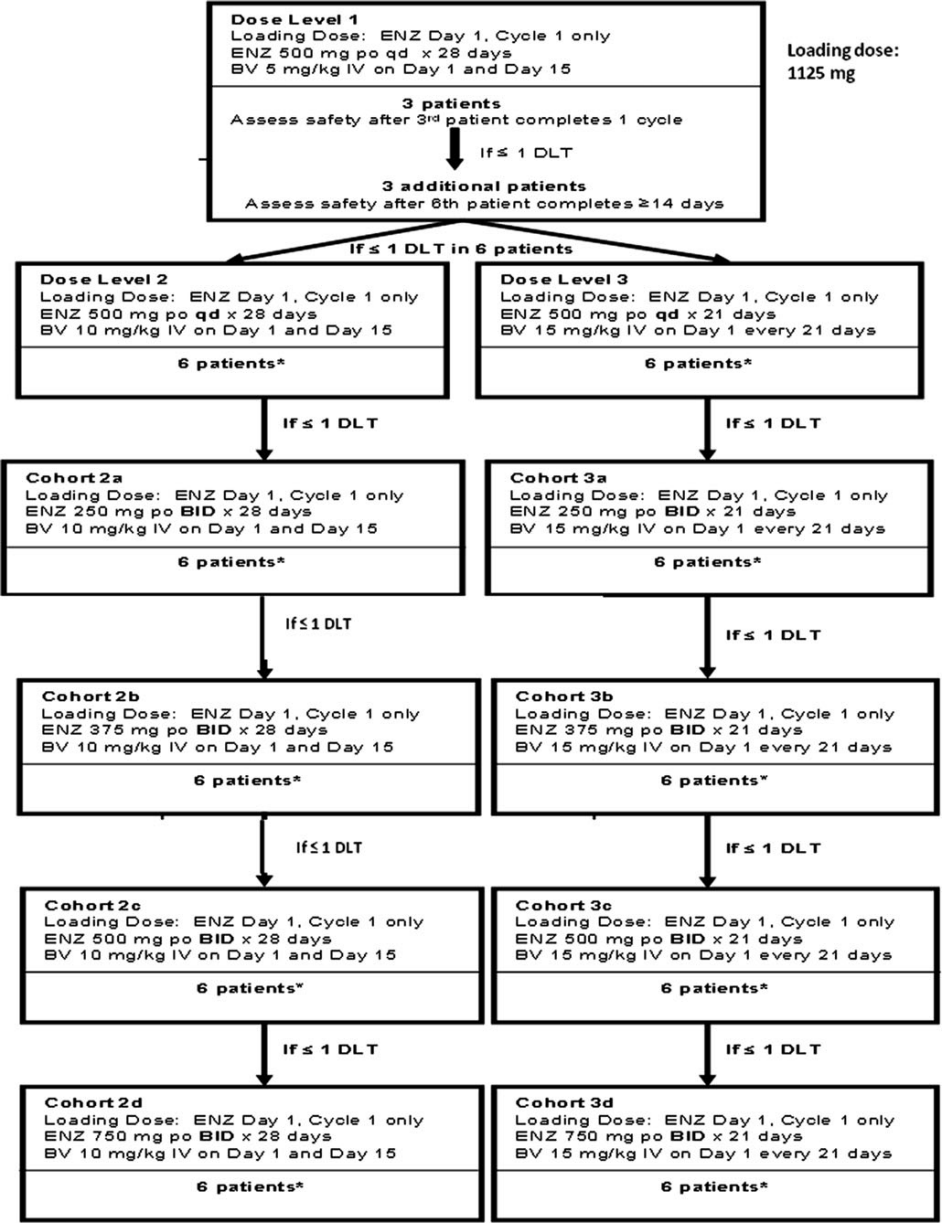
Study design. Dosing of the cohorts is shown. BID, twice daily; BV, bevacizumab; DLT, dose-limiting toxicity; ENZ, enzastaurin; IV, intravenous; PO, oral; QD, once daily. * All cohorts subsequent to Dose Level 1 followed the same enrollment pattern and safety assessment schedule shown in Dose Level 1

Figure 1 shows the study design. Each cohort enrolled 3 patients; if ≤1 dose-limiting toxicity (DLT) occurred, an additional 3 patients were enrolled in that cohort and dose escalation continued. The maximum tolerated dose (MTD) was achieved when 2 DLTs occurred in any given dose level; dose escalation then ceased, and the prior dose level was defined as the RP2D of the combination.

All patients continued on study drug therapy until progressive disease (PD), unacceptable toxicity, or other discontinuation criterion emerged. Once discontinued, patients were followed for 30 days following their last enzastaurin dose, or until they received another antitumor therapy.

### Patient evaluations

Adverse events (AEs) were assessed using National Cancer Institute Common Terminology Criteria for Adverse Events (NCI CTCAE version 3.0) criteria. Hematologic DLTs were: grade 4 neutropenia for ≥7 days; febrile neutropenia; and grade 3 thrombocytopenia with bleeding or grade 4 thrombocytopenia. Nonhematologic DLTs were: grade 3 or grade 4 toxicities; grade 4 hypertension; uncontrollable grade 3 hypertension; grade 4 hemorrhage; hemorrhage requiring discontinuation; any arterial thromboembolic event; grade 4 venous thrombosis; congestive heart failure; grade 4 proteinuria; gastrointestinal perforation, leak or fistula; grade 3 or grade 4 bowel obstruction; wound dehiscence requiring intervention; and grade 3 or 4 anaphylactic reactions to bevacizumab.

Tumor responses were assessed using RECIST guidelines [11]. Lesion assessments were repeated using the same methods as baseline. Tumor assessments by physical examination were performed prior to each cycle, whereas radiologic tumor assessments were done prior to every other cycle.

### Pharmacokinetic methods

Plasma concentration-time data from intensive sampling were used to calculate PK parameters for enzastaurin and the major active metabolite LY326020 on cycle 2 day 1 and bevacizumab on cycle 1 day 1. Enzastaurin and LY326020 samples were analyzed at Advion BioServices, Inc. (Ithaca, NY) using validated tandem liquid chromatography mass spectrometry. Bevacizumab samples were analyzed at Intertek (ALTA Analytical Laboratory, San Diego, CA) using a validated enzyme-linked immunosorbent assay (ELISA).

Standard non-compartmental methods (WinNonlin® Enterprise, Version 5.3) were used to calculate PK parameters.

The average concentration at steady state (C_av,ss_) was determined for enzastaurin and LY326020. The area under the plasma concentration–versus-time curve from time zero to infinity (AUC_[0-∞]_) was determined for bevacizumab.

### Biomarker (GSK3-β) analysis

Levels of pGSK3-β in peripheral blood mononuclear cells (PBMCs) were measured using an ELISA assay by Millipore (Billerica, MA). The pGSK3-β levels were normalized by the protein content of the sample.

### Statistical analysis

Evaluation of RP2D was conducted for all patients receiving ≥1 cycle of study medication and for patients who did not complete 1 cycle but experienced a DLT. Safety and PK analyses were performed for all patients receiving ≥1 dose of study drug. Antitumor activity was described using best overall response and time to tumor progression (TTP). Kaplan-Meier survival functions were estimated. Changes in pGSK3-β level from baseline were explored using a mixed-model repeated measures (MMRM) analysis with unstructured covariance to account for the within-patient correlation.

## Results

### Patient characteristics

From January 2007 to August 2009, 67 patients at Johns Hopkins Kimmel Cancer Center received ≥1 dose of study drug. Table 1 shows baseline demographics.

### Safety

The median delivered enzastaurin dose was 698 mg/d (range, 464 to 1938 mg/d); this was 99 % (range, 16 % to 100 %) of the planned dose. The median bevacizumab dose was 10 mg/kg (range, 5 to 19); this was 100 % of the planned dose. The mean ± standard deviation number of received cycles was 8 ± 10.

Six patients experienced 1 DLT each during cycle 1. The DLTs were grade 3 fatigue (*n* = 4; cohorts 2c, 2d, 3d), grade 4 cerebral hemorrhage (*n* = 1; cohort 2b), and grade 3 elevated aspartate transaminase at dose level 2 (*n* = 1). Two patients in cohort 2d experienced DLTs (grade 3 fatigue), thus defining the MTD. Consequently, the RP2 doses of enzastaurin were all preceding dose levels: 500 mg QD or 250, 375, or 500 mg BID, with bevacizumab 5 mg/kg or 10 mg/kg every 14 days, or 15 mg/kg every 21 days.

Eleven patients (16.4 %) experienced ≥1 serious adverse event (SAE) that was considered drug-related (see Online Resource 1, footnote b). At least 66 patients experienced a drug-related AE. Drug-related AEs (all grades) occurring in >20 % of patients were: urine color change (86.6 %), fatigue (68.7 %), gastrointestinal - other (52.2 %; mostly fecal discoloration), diarrhea (49.3 %), pain (47.8 %), nausea (41.8 %), pulmonary/upper respiratory hemorrhage (31.3 %), constipation (29.9 %), anorexia (26.9 %), abdominal distention/bloating (23.9 %), and vomiting (20.9 %). Online Resource 1 shows drug-related grades 3 through 5 AEs.

Fourteen patients discontinued due to AEs, of which 12 were serious. Nine discontinuations were from drug-related AEs, of which 7 were serious (grade 3 elevated aspartate transaminase and alanine transaminase; grade 4 cerebral hemorrhage; grade 3 failure to thrive; grade 4 genital tract fistula; grade 4 myocardial infarction; grade 3 thrombosis; and grade 3 pulmonary embolism). Each of the AEs and SAEs causing discontinuation occurred once. Six patients died during the trial; 2 deaths occurred on treatment, and 4 occurred within 30 days of treatment cessation. Of the 2 on-treatment deaths, 1 was from PD (cycle 4), and the second (ventricular tachycardia; cycle 1) was retrospectively considered possibly drug-related and confounded by PD. This patient with spinal involvement and potentially brain metastases, experienced a seizure-like situation and cardiac arrest with ventricular tachycardia on ECG. This event was not considered a DLT because the patient’s disease had progressed, and death more likely stemmed from central nervous system involvement rather than from study drug. Of the 4 deaths occurring after treatment, 1 was thought to be bevacizumab-related (cerebral hemorrhage; cycle 1), and 3 were due to PD.

### Pharmacokinetics

Table 2 shows the enzastaurin, LY326020, and bevacizumab PK results. When enzastaurin was administered as 500 mg daily, the mean C_av,ss_ of enzastaurin and LY326020 were similar across bevacizumab schedules. A dose-dependent increase in the enzastaurin mean C_av,ss_ was seen across the 250- to 750-mg BID dose range when enzastaurin was given with 10 or 15 mg/kg bevacizumab.

The mean AUC_(0-∞)_ for patients receiving bevacizumab 10 mg/kg ranged from 1740 μg·day/mL to 2360 μg·day/mL,

### Efficacy

Of the 67 enrolled patients, 19.4 % (95 % confidence interval [CI], 10.8 % to 30.9 %) responded to treatment (complete response, 9 %; partial response, 10.4 %) (Table 3). Complete responses were experienced by patients with ovarian (*n* = 4), esophageal (*n* = 1), and neuroendocrine tumors (*n* = 1), and partial responses were experienced by patients with ovarian (*n* = 6) and uterine papillary (*n* = 1) cancers.

Table 3 also shows measured time to event parameters in the entire cohort and ovcar subgroup per dose level.

### GSK3-β analysis

PBMC samples from 54 treated patients were evaluable for pGSK3-β. Online Resource 2 shows estimated mean pGSK3-β over time by dose schedule, which suggests a decreasing trend of pGSK3-β from baseline in both QD and BID schedules. However, the MMRM analysis did not suggest a statistically significant difference in pGSK-β decline over time between the 2 dosing schedules.

## Discussion

To determine the RP2D of enzastaurin and bevacizumab, this trial evaluated several dosing and scheduling regimens. Enzastaurin BID dosing was investigated because this schedule modestly increases exposures relative to QD dosing without clinically significant worsening of toxicities in most patients [6]. Oral enzastaurin (500 mg QD or 250, 375, or 500 mg BID), together with bevacizumab (5 mg/kg or 10 mg/kg every 14 days, or 15 mg/kg every 21 days) are well tolerated. The highest enzastaurin dose (750 mg BID) resulted in 4 DLTs (severe fatigue), with 2 occurring in cohort 2d (Fig 1), thus defining the MTD.

The combination of enzastaurin and bevacizumab did not appear to alter or exacerbate the AE profiles that have been observed when either drug was given alone. The majority of enzastaurin-related AEs observed here were consistent with those observed in another enzastaurin monotherapy study [5]. Other AEs were consistent with previous observations for bevacizumab [12].

The enzastaurin and LY326020 mean C_av,ss_ are similar to historical estimates for 250 mg BID (500 mg/d) and 500 mg QD [7, 9]. This study showed no evidence that the C_av,ss_ of enzastaurin or LY326020 were affected by bevacizumab (5, 10, or 15 mg/kg). Enzastaurinmean C_av,ss_ increases in a dose-dependent fashion when enzastaurin is dosed from 250 mg to 750 mg BID.

Although AUC_(0-∞)_ reported here for bevacizumab appears lower than in 2 historical studies [13, 14], no PK interaction is anticipated between enzastaurin and bevacizumab. Enzastaurin is primarily metabolized by CYP3A [5], whereas bevacizumab, a monoclonal antibody, is likely metabolized by the reticuloendothelial system[15]. The current study did not have a bevacizumab-only arm, so it is unknown whether the apparent difference in mean AUC_(0-∞)_ is due to enzastaurin co-administration or simply a consequence of interstudy variability.

The response rate in this trial was higher than that reported in our previous phase I trial involving patients with advanced cancer [5]. Patients receiving BID treatment experienced a higher rate of disease stabilization than those receiving QD treatment, and patients with ovcar seemed to fare better than expected.

The combination of enzastaurin and bevacizumab was previously tested in a phase II trial involving patients with recurrent glioblastoma [16]. Although the combination was well tolerated and exhibited clinical activity, it did not appear to improve clinical outcomes relative to bevacizumab monotherapy. In contrast, the combination of enzastaurin and bevacizumab therapy appears to have delayed disease progression in the ovcar subset relative to results published for bevacizumab monotherapy [10, 17] and 500 mg QD enzastaurin monotherapy in patients with ovcar [18]. This suggests that the combination may have a unique effect on ovarian tumors; however, it should be noted that the BID dosing schedule of enzastaurin may have provided an exposure advantage relative to the glioblastoma study, which used QD dosing [16]. The promising results for the ovcar subset should be viewed cautiously due to the small number of heterogeneous patients. Thus, further confirmatory studies are needed.

The activity of GSK3-β is inhibited by PKB/AKT-dependent phosphorylation [19] and thus, is a potential biomarker of enzastaurin activity [2]. On average, pGSK3-β levels were decreased from baseline in both schedules, with a maximum occurring at treatment discontinuation. However, no statistically significant difference in mean pGSK3-β across time was found possibly related to large interpatient variability. The decline of pGSK3-β levels with enzastaurin exposure time is consistent with previous findings, suggesting that the pGSK3-β inhibitory effects in PBMCs are a component of the antitumor activity of enzastaurin [2]. The pGSK3-β results are exploratory in nature and warrant further confirmatory studies.

In conclusion, this study showed no evidence that the PK of enzastaurin or LY326020 were affected when enzastaurin was administered with bevacizumab (5, 10, or 15 mg/kg). The observed antitumor activity in patients receiving both enzastaurin and bevacizumab was higher than that reported in our previous phase I trial involving patients with advanced cancer [5], with outcomes (median duration of stable disease; median TTP) appearing to favor the BID enzastaurin dosing groups. Combination therapy showed limited but potentially encouraging efficacy results in ovcar patients, but we cannot exclude the possibility that the observed activity is due to bevacizumab alone. The observed benefit should be confirmed in subsequent controlled trials and suggests that PI3K/AKT inhibition in combination with anti-VEGF therapy may be worth pursuing in patients with ovcar.

## Electronic supplementary material

Below is the link to the electronic supplementary material.

Online Resource 1 (PDF 133 kb)

Online Resource 2 (PDF 54 kb)
