# Supplementary material for: Phase I study of enzastaurin and bevacizumab in patients with advanced cancer: safety, efficacy and pharmacokinetics
Source: Invest New Drugs. 2012 Jul 6;31(3):653–60. doi: 10.1007/s10637-012-9850-6 (PMC3644404; doi:10.1007/s10637-012-9850-6)
Supplement: Supplementary file 1 — (PDF 133 kb) [file 10637_2012_9850_MOESM1_ESM.pdf]

Online Resource 1. CTCAE Grades 3 through 5 Drug-Related Adverse Events by Dose Cohort[a]

CTCAE Term	DL 1 n=6	DL 2 n=6	DL 3 n=6	DL 2a n=6	DL 3a n=6	DL 2b n=6	DL 3b n=7	DL 2c n=8	DL 3c n=7	DL 2d n=3	DL 3d n=6	Total N=67
**Laboratory, n (%)**												
Patients with ≥1Grades 3 through 5 Laboratory CTCAE	0 (0.0)	**1 (16.7)**	**1 (16.7)**	0 (0.0)	0 (0.0)	0 (0.0)	0 (0.0)	0 (0.0)	**1 (14.3)**	0 (0.0)	**1 (16.7)**	**4 (6.0)**
ALT/SGPT (grade 3)	0 (0.0)	0 (0.0)	0 (0.0)	0 (0.0)	0 (0.0)	0 (0.0)	0 (0.0)	0 (0.0)	0 (0.0)	0 (0.0)	**1 (16.7)**[b]	**1 (1.5)**
AST/SGOT (grade 3)	0 (0.0)	**1 (16.7)**	0 (0.0)	0 (0.0)	0 (0.0)	0 (0.0)	0 (0.0)	0 (0.0)	**1 (14.3)**	0 (0.0)	**1 (16.7)**[b]	**3 (4.5)**
Platelets (grade 3)	0 (0.0)	0 (0.0)	**1 (16.7)**	0 (0.0)	0 (0.0)	0 (0.0)	0 (0.0)	0 (0.0)	0 (0.0)	0 (0.0)	0 (0.0)	**1 (1.5)**
**Non-Laboratory, n (%)**												
Patients with ≥1 Grades 3 through 5 Non-Laboratory CTCAE	**1 (16.7)**	0 (0.0)	**3 (50.0)**	**1 (16.7)**	0 (0.0)	**2 (33.3)**	0 (0.)	**4 (50.0)**	**3 (42.9)**	**3 (100)**	**3 (50.0)**	**20 (29.9)**
Anorexia (grade 3)	0 (0.0)	0 (0.0)	0 (0.0)	0 (0.0)	0 (0.0)	0 (0.0)	0 (0.0)	**1 (12.5)**	0 (0.0)	0 (0.0)	0 (0.0)	**1 (1.5)**
Cardiac (grade 5)	0 (0.0)	0 (0.0)	0 (0.0)	0 (0.0)	0 (0.0)	0 (0.0)	0 (0.0)	0 (0.0)	0 (0.0)	**1 (33.3)**[b,d]	0 (0.0)	**1 (1.5)**
Cardiac infarction (grade 4)	0 (0.0)	0 (0.0)	**1 (16.7)**[b]	0 (0.0)	0 (0.0)	0 (0.0)	0 (0.0)	0 (0.0)	0 (0.0)	0 (0.0)	0 (0.0)	**1 (1.5)**
Constitutional symptoms (grade 3)	0 (0.0)	0 (0.0)	0 (0.0)	0 (0.0)	0 (0.0)	0 (0.0)	0 (0.0)	**2 (25.0)**	0 (0.0)	0 (0.0)	0 (0.0)	**2 (3.0)**
Asthenia (grade 3)	0 (0.0)	0 (0.0)	**1 (16.7)**	0 (0.0)	0 (0.0)	**1 (16.7)**	0 (0.0)	**2 (25.0)**[b]	**1 (14.3)**	**3 (100.0)**	**1 (16.7)**	**9 (13.4)**
Fistula, genitourinary (grade 4)	0 (0.0)	0 (0.0)	0 (0.0)	**1 (16.7)**[b]	0 (0.0)	0 (0.0)	0 (0.0)	0 (0.0)	0 (0.0)	0 (0.0)	0 (0.0)	**1 (1.5)**
Hemorrhage, CNS (grade 5)	0 (0.0)	0 (0.0)	0 (0.0)	0 (0.0)	0 (0.0)	**1 (16.7)**[b]	0 (0.0)	0 (0.0)	0 (0.0)	0 (0.0)	0 (0.0)	**1 (1.5)**
Infection with grade 3/4 neutrophils (grade 3)	0 (0.0)	0 (0.0)	0 (0.0)	0 (0.0)	0 (0.0)	0 (0.0)	0 (0.0)	0 (0.0)	0 (0.0)	0 (0.0)	**1 (16.7)**	**1 (1.5)**
Infection with unknown ANC (grade 3)	0 (0.0)	0 (0.0)	0 (0.0)	0 (0.0)	0 (0.0)	0 (0.0)	0 (0.0)	0 (0.0)	**1 (14.3)**[b,c]	0 (0.0)	0 (0.0)	**1 (1.5)**
Pain (grade 3)	**1 (16.7)**	0 (0.0)	0 (0.0)	0 (0.0)	0 (0.0)	0 (0.0)	0 (0.0)	0 (0.0)	0 (0.0)	0 (0.0)	0 (0.0)	**1 (1.5)**
Pancreatitis (grade 3)	0 (0.0)	0 (0.0)	0 (0.0)	0 (0.0)	0 (0.0)	0 (0.0)	0 (0.0)	0 (0.0)	**1 (14.3)**[b]	0 (0.0)	0 (0.0)	**1 (1.5)**
Prolonged QTc interval (grade 3)	0 (0.0)	0 (0.0)	0 (0.0)	0 (0.0)	0 (0.0)	0 (0.0)	0 (0.0)	0 (0.0)	0 (0.0)	0 (0.0)	**1 (16.7)**	**1 (1.5)**
Rash/desquamation (grade 3)	0 (0.0)	0 (0.0)	0 (0.0)	0 (0.0)	0 (0.0)	0 (0.0)	0 (0.0)	**1 (12.5)**	0 (0.0)	0 (0.0)	0 (0.0)	**1 (1.5)**
Somnolence (grade 4)	0 (0.0)	0 (0.0)	0 (0.0)	0 (0.0)	0 (0.0)	0 (0.0)	0 (0.0)	0 (0.0)	0 (0.0)	**1 (33.3)**	0 (0.0)	**1 (1.5)**

Online Resource 1. CTCAE Grades 3 through 5 Drug-Related Adverse Events by Dose Cohort[a]

CTCAE Term	DL 1 n=6	DL 2 n=6	DL 3 n=6	DL 2a n=6	DL 3a n=6	DL 2b n=6	DL 3b n=7	DL 2c n=8	DL 3c n=7	DL 2d n=3	DL 3d n=6	Total N=67
Thrombosis (grade 3)	0 (0.0)	0 (0.0)	**1 (16.7)**[b]	0 (0.0)	0 (0.0)	0 (0.0)	0 (0.0)	0 (0.0)	0 (0.0)	0 (0.0)	**1 (16.7)**[b]	**2 (3.0)**
Vomiting (grade 3)	0 (0.0)	0 (0.0)	0 (0.0)	0 (0.0)	0 (0.0)	0 (0.0)	0 (0.0)	0 (0.0)	**1 (14.3)**[b]	0 (0.0)	0 (0.0)	**1 (1.5)**

Abbreviations: ALT/SGPT, alanine transaminase/glutamic pyruvic transaminase; ANC, absolute neutrophil count; AST/SGOT, aspartate transaminase/glutamic oxaloacetic transaminase; CNS, central nervous system; CTCAE, Common Terminology Criteria for Adverse Events; SAE, serious adverse event.

[a] On study or within 30 days of discontinuation.

[b] Indicates an SAE coded using the MedDRA Version 9.0 dictionary. Other SAEs are: Dose Level 2c, failure to thrive; Dose Level 3d, pulmonary embolism.

[c] Postoperative wound infection.

[d] Ventricular tachycardia.
