# Supplementary material for: Phase I study of enzastaurin and bevacizumab in patients with advanced cancer: safety, efficacy and pharmacokinetics
Source: Invest New Drugs. 2012 Jul 6;31(3):653–60. doi: 10.1007/s10637-012-9850-6 (PMC3644404; doi:10.1007/s10637-012-9850-6)
Supplement: Supplementary file 2 — (PDF 54 kb) [file 10637_2012_9850_MOESM2_ESM.pdf]

Online Resource 2.

MMRM Analyses of pGSK3-beta					
Dose Schedule	Sample Number	N	LSMean	SE	*P*[a]
QD	C1D1, 4 hrs	12	2.28	0.26	.473
BID	C1D1, 4 hrs	40	2.08	0.15	
QD	C1D2, 24 hrs	11	2.06	0.23	.366
BID	C1D2, 24 hrs	31	1.84	0.13	
QD	C2D1, pre-dose	7	1.85	0.26	.384
BID	C2D1, pre-dose	27	1.59	0.15	
QD	Discontinuation	6	1.63	0.33	.464
BID	Discontinuation	14	1.35	0.20	

Abbreviations: BID, twice daily; C, cycle; D, day; LSMean, least-square mean; MMRM = mixed-model analysis of repeated measures; QD = once daily; SE = standard error.

[a] Between-group comparison.

Note: pGSK3-beta values are natural log transformed prior to MMRM analyses.
